# Changes in Coping Strategies of Parents and Girls with Central Precocious Puberty Before and After the COVID-19 Lockdown: Data from Four Italian Pediatric Endocrinology Centers

**DOI:** 10.3390/ijerph22070981

**Published:** 2025-06-21

**Authors:** Maria E. Street, Anna-Mariia Shulhai, Dolores Rollo, Maurizio Rossi, Maddalena Petraroli, Vittorio Ferrari, Giulia Del Medico, Patrizia Bruzzi, Beatrice Righi, Chiara Sartori, Lorenzo Iughetti, Stefano Stagi

**Affiliations:** 1 Department of Medicine and Surgery, University of Parma, 43121 Parma, Italy; mariaelisabeth.street@unipr.it (M.E.S.); annashulhai@gmail.com (A.-M.S.); maurizio.rossi@unipr.it (M.R.); 2 Unit of Paediatrics, P. Barilla Children’s Hospital, University Hospital of Parma, 43121 Parma, Italy; mpetraroli@ao.pr.it; 3 Pediatric Unit, IRCCS Azienda Ospedaliero-Universitaria di Bologna, 40138 Bologna, Italy; vittorioferrari15@gmail.com; 4 Department of Medical and Surgical Sciences, University of Bologna, 40138 Bologna, Italy; 5 Department of Health Sciences, University of Florence, 50139 Florence, Italy; giulia.delmedico@unifi.it (G.D.M.); stefano.stagi@unifi.it (S.S.); 6 Pediatric Unit, Department of Medical and Surgical Sciences for Mother Children and Adults, University of Modena and Reggio Emilia, 41124 Modena, Italy; patrizia.bruzzi@unimore.it (P.B.); iughetti.lorenzo@unimore.it (L.I.); 7 Unit of Paediatrics, AUSL-IRCCS di Reggio Emilia, 42122 Reggio Emilia, Italy; beatrice.righi2406@gmail.com (B.R.); chiara.sartori@ausl.re.it (C.S.); 8 Meyer Children’s Hospital IRCCS, 50139 Florence, Italy

**Keywords:** central precocious puberty, CPP, COVID-19, coping strategies, psychosocial stress

## Abstract

The increased stress during the COVID-19 pandemic may have influenced the coping strategies used by children and parents who adapted to a diagnosis of central precocious puberty (CPP). This study aimed to explore whether the coping mechanisms of parents and their daughters diagnosed with CPP differed before and after the COVID-19 lockdown and if certain factors could be associated with these mechanisms. Specific questionnaires were completed by 174/524 girls with CPP enrolled at four different pediatric endocrinology centers in Italy. All girls filled in the questionnaire about the Children’s Coping Strategies (CCSs), and their parents completed the Coping Orientation to the Problems Experienced (COPE-NVI-25) questionnaire. Cronbach’s test was performed to check the reliability of answers. Despite increased stress-related coping behaviors among girls with CPP after the lockdown, parents presented more functional coping behaviors related to problem-solving strategies and seeking social support. After the lockdown, children showed stronger associations with their parents’ adaptive strategies, emphasizing the important role of parental behaviors in shaping the responses of children to stressful events. Additionally, coping strategies were found to differ by country of origin: families of non-Italian origin showed a greater reliance on transcendence, while girls from these families had lower avoidance scores, suggesting the use of alternative adaptive strategies in stressful situations.

## 1. Introduction

Puberty is the stage of life that leads to adulthood through significant physical and psychological changes and is characterized by the development of secondary sexual characteristics [1]. Central precocious puberty (CPP) is characterized by the early onset of puberty, typically before the age of 8 in girls and 9 in boys, because of the premature activation of the hypothalamic–pituitary–gonadal axis (HPG), leading to the early release of sex hormones and the appearance of secondary sexual characteristics [2,3]. Early diagnosis and intervention are essential to reduce potential long-term consequences, such as reduced adult height and psychosocial issues [4].

Coping with the physical changes and emotional challenges associated with CPP can be overwhelming for children and their families and may have worsened during the COVID-19 pandemic due to significant lifestyle changes, schedules, and school modalities.

While stress mediators typically inhibit the reproductive axis in adults, prolonged activation of the hypothalamic–pituitary–adrenal axis in prepubertal individuals may lead to early puberty onset [5]. Thus, the lifestyle changes and emotional stress caused by the COVID-19 pandemic may be among the causes of the increased prevalence of CPP in girls [6,7].

Coping strategies, defined as the cognitive and behavioral efforts to manage stressful situations, can shape how children and their caregivers adapt to the diagnosis and progression of CPP. Studying these strategies is important not only to understand the psychological impact of CPP but also to identify potential intervention targets to improve psychological adjustment. However, few studies have directly examined how children with CPP and their parents cope with this condition [8], particularly within the context of the pandemic.

We hypothesized that analyzing the coping mechanisms employed by girls diagnosed with CPP and their parents could provide insights into their psychological adaptation, especially when comparing those assessed before and after the COVID-19 lockdown.

Therefore, the aim of this study was to explore whether the COVID-19 lockdown might have changed the coping mechanisms of both parents and their daughters diagnosed with CPP. The use of validated instruments to assess coping strategies in both children and adults allows for a structured analysis of psychological responses to the diagnosis and its associated stressors. Additionally, in a substudy, we analyzed the impact of other factors, like adoption, geographical origins, and family history of precocious puberty, on coping strategies before and after the lockdown related to the pandemic. These factors have been implicated in previous literature as capable to influence on the psychosocial outcomes of both pubertal development and stress adaptation [5].

## 2. Materials and Methods

### 2.1. Participants

This multicenter retrospective study included 174/524 girls diagnosed with central precocious puberty and their parents, enrolled at four pediatric endocrinology centers in Italy: the University Hospital in Parma, the Meyer’s Children’s Hospital IRCCS in Florence, the University Hospital in Modena, and the Hospital in Reggio Emilia. Participants were enrolled either before February 2020 or after May 2020, i.e., the Italian COVID-19 lockdown.

All participant girls were diagnosed with CPP and underwent a physical examination and auxological evaluation. Weight was measured using an electronic scale with a weight accuracy of 50 g. Height was measured using a Harpenden Stadiometer in triplicate to the nearest 0.1 cm. Body mass index (BMI) was calculated for all children according to the formula by dividing the patient’s weight in kilograms by the square of height in meters. Height and BMI were normalized for chronological age by conversion to standard deviation (SD) scores using Italian reference data [9]. Target height was calculated based on sex-adjusted mid-parental height [father’s height + mother’s height − 13]/2 and then converted to SDS [10]. Pubertal stages were classified according to the Marshall and Tanner criteria [11]. The age of pubertal onset was defined as the age at the durable Tanner B2 stage [11]. 

A detailed patient history was taken for all subjects (geographical origin (Italian vs. non-Italian), adoption, mother’s age at menarche, father’s pubertal development, and presence or absence of family history of precocious puberty).

Blood samples for baseline biochemical assessment were taken after an overnight fast. Hormonal assessment of luteinizing hormone (LH), follicle-stimulating hormone (FSH), estradiol (E2), dehydroepiandrosterone sulfate (DHEAS), 17-hydroxyprogesterone (17-OHP), prolactin (PRL), thyroid-stimulating hormone (TSH), free thyroxine (fT4), and the GnRH stimulation test were performed in all patients.

Central precocious puberty was defined as breast development, with or without axillary/pubic hair before the age of 8; a baseline LH level of >1.1 IU/L together with pubertal signs; or a GnRH-stimulated peak LH level of >5 IU/L, with an LH/FSH peak ratio of >1.0 combined with isolated and/or axillary hair growth accompanied by breast development [12].

Transabdominal pelvic ultrasonography was used to evaluate the shape and size of the uterus and ovaries, and whenever it was available, endometrial thickness and uterine body-to-cervix ratio were defined.

Bone age (BA) assessment using radiographs of the non-dominant hand was performed. Bone age was assessed using the Greulich and Pyle method [13]. Then, the difference between years, chronological age–bone age (Delta CA–BA), and the ratio of the change in BA to the change in chronological age (CA) (BA/CA) were calculated.

Brain magnetic resonance imaging (MRI) of the hypothalamus and pituitary gland was performed in for patients.

The inclusion criteria for this study were females, born from 2009 onward, confirmed CPP diagnosis via GnRH stimulus test and MRI scan of the brain, full-filled questionnaires for Children’s Coping Strategies (CCSs) for the participants and Coping Orientation to Problems Experienced (COPE-NVI-25) questionnaires for their parents, and obtained informed consent. The exclusion criteria were unconfirmed CPP; CPP associated with hypothalamic–pituitary congenital malformations; neurological, neurosurgical, and/or genetic diseases; oncological diseases; and children on medication that could interfere with pubertal development.

### 2.2. Specific Questionnaires

All participating girls and their parents completed questionnaires to assess coping strategies. The participants filled in the questionnaire about Children’s Coping Strategies (CCSs) [14], and their parents completed the Coping Orientation to Problems Experienced (COPE-NVI-25) questionnaire [15].

The Children’s Coping Strategies (CCSs) is a validated questionnaire to assess coping strategies for stressful events implemented by children [14]. It consists of 18 questions grouped into three items: avoidance, coping and support-seeking, and aggressive behaviors. Each of these categories represents a coping strategy to a stressful event. Some strategies are more common and functional (coping and support-seeking), while others are dysfunctional (avoidance and aggressive behaviors). Each of the three items has a variable number of subitems, which, taken individually, define behaviors belonging to the same coping strategy. Each subitem is assigned a score from 1 to 4, based on the frequency of implementation of a given behavioral pattern: 1 = Never; 2 = Rarely; 3 = Sometimes; 4 = Often.

For the purpose of this study, scores for each of the three coping strategies (avoidance, coping and support-seeking, and aggressive behaviors) were calculated as the mean of their respective subitems. Higher mean scores indicate a greater frequency of using that specific coping strategy. These mean scores were treated as continuous variables in our analyses. Functional coping was operationally defined by higher scores on the coping and support-seeking scale, while dysfunctional coping was indicated by higher scores on the avoidance and aggressive behaviors scales. The CCS has shown good internal consistency acceptable to good reliability coefficients (from 0.77 to 0.92) [14].

The COPE-NVI-25 questionnaire represents a simplified version of the Coping Orientation to Problem Experienced-New Italian Version (COPE-NVI) [15]. The COPE-NVI-25 consists of 5 items and 25 subitems, each of which parents were asked to score from 1 to 6, where 1 means “not true at all” and 6 means “completely true.” Each item describes a different macro coping strategy. Assessing the frequency of individual strategies provides an accurate and comprehensive measure of how people cope with stressful events. Functional coping strategies include a positive attitude, seeking social support, and problem-focused orientation items, and overall describe a problem-focused coping style. In contrast, dysfunctional strategies correspond to avoidance and transcendent orientation and describe an emotion-focused coping style.

Scores for each of the five coping strategies (positive attitude, seeking social support, problem-focused orientation, avoidance, and transcendent orientation) were derived by calculating the mean of their corresponding subitems. These mean scores were used as continuous variables in our analyses, with higher scores indicating greater reliance on that specific coping strategy. Functional coping was operationalized by higher mean scores on the positive attitude, seeking social support, and problem-focused orientation scales. Dysfunctional coping was operationalized by higher mean scores on the avoidance and transcendent orientation scales. The COPE-NVI-25 has demonstrated good and excellent psychometric properties in the Italian population, with subscale Cronbach’s alpha values ranging between 0.70 and 0.96, and evidence of factorial validity [15]. It has been used in various contexts, including health-related and parental stress research, and is suitable for assessing coping mechanisms in parents facing their child’s health challenges.

The questionnaires were sent to the parents of the participants via e-mail or were delivered in person during follow-up outpatient visits at the pediatric endocrinology centers in the cities participating in this study. After signing informed consent to this study, the referring parent (mother in most cases) and the daughter were asked to fill in the questionnaires, making an effort to mentally return to the time of the onset of their daughter’s first pubertal signs and symptoms. Thirty-nine girls were diagnosed with idiopathic CPP before February 2020 (the beginning of the lockdown), and 135 were diagnosed during/after February 2020 (post-lockdown).

### 2.3. Ethical Committee Approval

This study was conducted according to the Helsinki II declaration and was approved by the Paediatric Ethical Committee AVEN (approval number: 365/2022/OSS/UNIPR PP.COVID-19) and by the ethical committee at the Meyer Hospital. Informed consent was obtained from the parents of all enrolled children.

### 2.4. Statistical Analysis

Statistical analysis was conducted using IBM SPSS Statistics ver. 22.0 (IBM SPSS Corp., Armonk, NY, USA), JASP ver. 0.17.1 (Intel, Amsterdam, The Netherlands), and Microsoft Excel (2013). The normality of the distribution of variables was evaluated using the Shapiro–Wilk test. As most of the parameters were normally distributed, continuous variables were shown as mean (M) and standard deviation (SD). Categorical variables were analyzed in terms of absolute frequencies and percentages. A comparison of two independent samples with normal distribution was performed using Student’s t-test, and comparisons of two independent samples with non-normal distribution of values were performed using Mann–Whitney’s nonparametric U-test. Pearson’s or Spearman’s correlation coefficients were used to evaluate possible associations between the studied data, depending on distribution. Correlations were considered significant if r > 0.300. Multiple linear regression analyses were used to investigate potential factors associated with responses to the COPE-NVI-25 and CCS questionnaires.

Cronbach’s alpha coefficients were calculated to assess the internal consistency (reliability) of the instruments used in this study. Separate values were computed for the CCS questionnaire and COPE-NVI-25 questionnaire to provide a clear and accurate evaluation of the psychometric properties of each instrument. For the CCS questionnaire, Cronbach’s alpha was α = 0.798, indicating acceptable reliability. For the COPE-NVI-25 questionnaire, Cronbach’s alpha was α = 0.874, indicating good internal consistency.

The significance of differences between values was considered significant at *p* < 0.05.

## 3. Results

### 3.1. Clinical Features

The main clinical, hormonal, and instrumental data of the 174 girls diagnosed with idiopathic CPP, comparing the pre- (before February 2020) and post-lockdown periods (after May 2020), are reported in Table 1. No significant differences were observed between the two groups of participants. However, LH, peak LH, and the LH/FSH ratio after GnRH stimulation were significantly higher in the girls diagnosed after the lockdown (*p* < 0.010).

**Figure 1:**
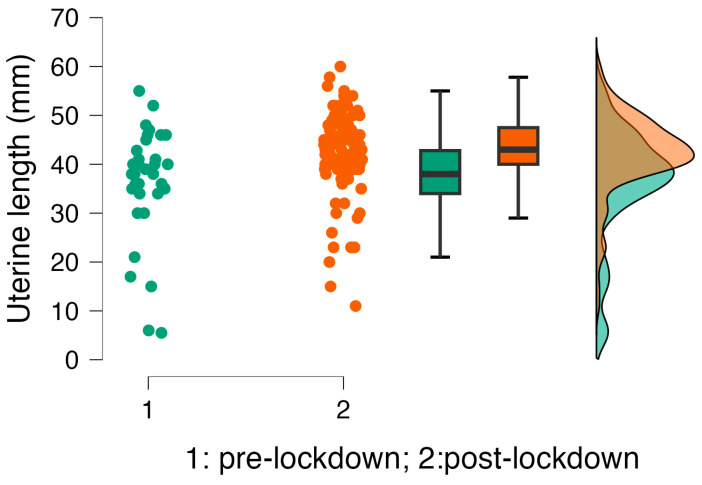
Comparison of uterine lengths in girls diagnosed with central precocious puberty in the pre- and post-lockdown periods. Green indicates the pre-lockdown period, and orange indicates the post-lockdown period.

Transabdominal pelvic ultrasonography showed differences in uterine lengths among girls diagnosed before and after the lockdown, with a significant increase in the post-lockdown group (Figure 1), suggesting a greater estradiol stimulation or a later diagnosis.

Correlation analysis showed a significant association between uterine length and basal LH levels (r = 0.340; *p* = <0.001), peak LH (r = 0.436; *p* = <0.001), LH/FSH ratio (r = 0.416; *p* = <0.001), and estradiol serum concentrations (r = 0.496; *p* = <0.001), as expected.

### 3.2. Coping Strategies

Coping strategies were evaluated by both parents and their daughters to explore any differences before and after the COVID-19 lockdown. The total scores for each item are reported in Table 2. There were no significant differences between the pre- and post-lockdown.

In both periods, children reported using “Coping and seeking support” strategies more frequently than “Avoidance” and “Aggressive behaviors.” Parents (COPE-NVI-25 questionnaire) reported mostly functional coping strategies (problem-focused orientation, positive attitude, and seeking social support) compared to dysfunctional coping strategies (avoidance and transcendent orientation), with no differences between the pre- and post-lockdown periods.

### 3.3. Correlation Analysis

Correlation analyses were conducted with an exploratory approach to investigate possible changes in the patterns of associations between coping strategies of parents and their daughters before and after the lockdown. However, these results should be interpreted with caution, as differences in correlation strength may reflect variations in sample size rather than true temporal effects.

**Figure 2:**
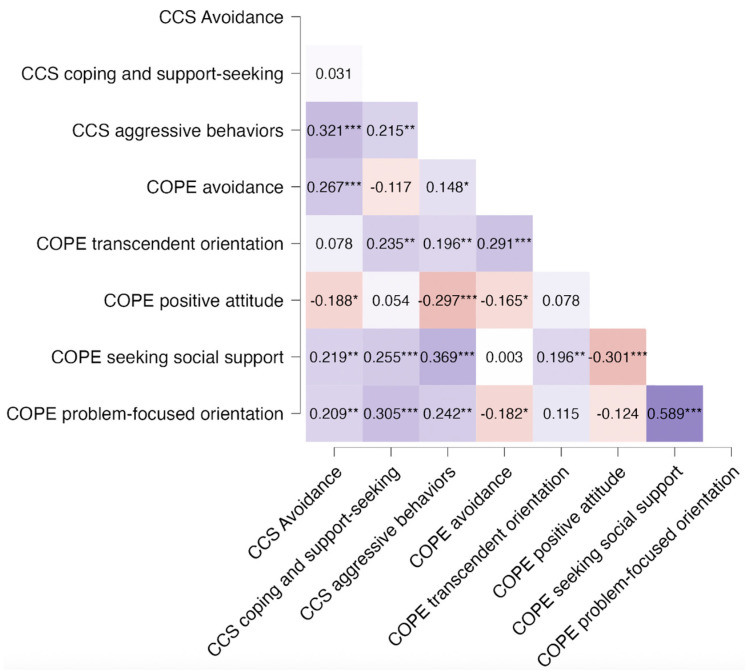
Heatmap of correlations among coping strategies of parents and children in the entire CPP group. Colors indicate the direction and strength of relationships, with blue representing positive correlations and red representing negative correlations. *** *p* < 0.001; ** *p* < 0.01; * *p* < 0.05.

In the entire group, associations among items from the COPE-NVI-25 and the CCS questionnaires are reported in Figure 2.

Among parents, “Problem Orientation” and “Seeking Social Support” (COPE-NVI-25) were positively associated (r = 0.589; *p* < 0.001), whereas “Positive attitude” and “Problem-focused orientation” were negatively correlated (r = −0.301, *p* < 0.001).

In the children, “Aggressive Behaviors” was significantly correlated with “Avoidance” (r = 0.321; *p* < 0.001) and “Seeking Social Support” in the parents (COPE) (r = 0.369; *p* < 0.001). “Coping and support-seeking” in the girls (CCS) was associated with COPE “Problem-focused orientation” in their parents (r = 0.305; *p* < 0.001).

**Figure 3:**
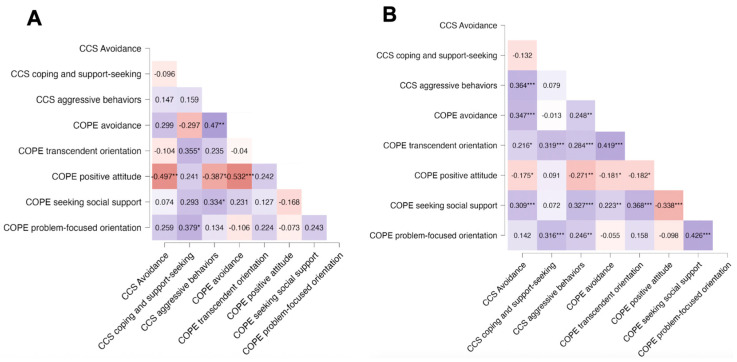
Heatmaps of correlations between items from the COPE-NVI-25 questionnaire completed by parents and items from the CCS questionnaire completed by their daughter, having CPP. (**A**) Before lockdown and (**B**) after the COVID-19 lockdown. Colors indicate the direction and strength of the relationships, with blue representing positive correlations and red negative correlations. *** *p* < 0.001; ** *p* < 0.01; * *p* < 0.05.

Correlations were subsequently analyzed for the two subgroups, before and after the lockdown, and are reported in Figure 3A and Figure 3B, respectively.

Before the lockdown, in parents, “Avoidance” was strongly negatively correlated with a “Positive attitude,” whereas after the lockdown, this association was weaker and became associated with a “Transcendent orientation.” Moreover, in the post-lockdown period, parents’ “Seeking Social Support” and “Problem-focused orientation” were significantly associated (r = 0.426, *p* < 0.001) with each other.

The girls did not show significant associations before the lockdown. However, a stronger positive correlation between “Avoidance” and “Aggressive Behaviors” was observed in the post-lockdown period compared to the pre-lockdown period.

Overall, in the pre-lockdown period, several relationships appeared stronger compared to the post-lockdown period, such as between CCS “Aggressive Behaviors” and COPE “Avoidance” (r = 0.47, *p* < 0.01 pre-lockdown vs. r = 0.248, *p* < 0.01 post-lockdown), and the negative association between COPE “Positive attitude” and CCS “Aggressive Behaviors” (r = −0.387, *p* < 0.05 pre-lockdown vs. r = −0.271, *p* < 0.01 post-lockdown) and CCS “Avoidance” (r = −0.497, *p* < 0.01 pre-lockdown vs. r = −0.175, *p* < 0.05 post-lockdown).

In the post-lockdown group, CCS “Coping and support-seeking” in girls remained significantly associated with COPE “Transcendent orientation” (r = 0.319, *p* < 0.001) and COPE “Problem-focused orientation” (r = 0.316, *p* < 0.001). CCS “Aggressive Behaviors” was observed to be significantly associated with COPE “Seeking Social Support” (r = 0.327, *p* < 0.001). “Avoidance” in girls shifted from a negative correlation with “Positive Attitude” in parents to a positive correlation with “Seeking Social Support” in parents (r = 0.309, *p* < 0.001).

To establish whether geographical origins of the families or other factors were associated with questionnaire responses, multiple regression analyses were performed in a subgroup of 44 girls with CPP for whom detailed family information was available. Non-Italian origin showed higher scores on the “Transcendent Orientation” (*p* = 0.041) in parents and on the “Avoidance” in the daughters (*p* = 0.002), where non-Italian girls had lower scores compared to Italian girls (Table 3). No differences were observed between adopted versus non-adopted girls, in those having or not having a family history of precocious puberty. The age at menarche of the mothers had no association.

## 4. Discussion

This study analyzed coping strategies adopted by children and their parents before and after the COVID-19 lockdown in relation to the diagnosis of central precocious puberty in girls. Our findings established shifts in coping behaviors, with both children and parents adopting more adaptive, problem-focused, and transcendent-oriented coping strategies in the post-lockdown period. Pre-lockdown, stronger associations were found between children’s aggressive behaviors and avoidance strategies in parents, and between parental positive attitudes and reduced aggressive behavior in the children. Post-lockdown, children’s behaviors showed stronger links with parental social support-seeking. Additionally, children’s coping and seeking social support were more associated with parents’ problem-focused and transcendent orientations. This suggests an adaptive shift towards more constructive coping mechanisms over time, possibly reflecting adjustments to prolonged stress exposure. While some shifts in the correlations between coping strategies were observed across time points, these findings should be interpreted as exploratory and not indicative of causal relationships.

In our study, we observed higher LH, LH peaks, and increased uterine lengths in girls diagnosed with CPP post-lockdown compared to pre-lockdown peers, with no changes in other clinical, biochemical, or instrumental parameters. This could be attributed to possible combined pathophysiological, emotional, and psychological stressors mentioned above, supported by previously published research [16,17,18].

Anthropometric, biochemical, and hormonal parameters, along with parental auxological data, did not affect the responses to the questionnaires. We did not observe significant changes in item scores of the CCS and COPE-NVI-25 questionnaires. Both children and parents showed predominant functional coping strategies during the pre- and post-lockdown periods. Correlation analysis of the entire group defined an association between the COPE items “Problem-focused orientation” and “Seeking Social Support.” This result suggests that parents who had a more problem-oriented approach to coping with their daughter’s diagnosis, using active and planning strategies more frequently, sought confrontation with and support from others [19].

Children had a strong association between avoidance strategies and aggressive behaviors. This suggests that rather than confronting stressors directly, they may respond with aggression while simultaneously withdrawing from stressful situations. Researchers have hypothesized that stress-related factors could be due to changes in daily functioning, treatment, or uncertainty [8,20]. However, “Aggressive Behaviors” in children were strongly associated with “Seeking Social Support” in parents. The increased emotional distress in children likely stimulates parents to seek external support. Furthermore, the positive association between problem orientation in parents and coping and support-seeking strategies in their daughters’ supports the fact that when parents adopt more functional approaches, their children are more likely to adopt the same. These findings emphasize the importance of parent–child interactions in shaping coping responses. Previous research has indicated that parental stress and coping mechanisms have been associated with children’s behavioral responses to adversity [21,22,23]. Therefore, family engagement may help reduce child symptoms of chronic illnesses [24] and increase child’s coping [20].

Comparing pre- and post-lockdown coping mechanisms showed both stability and shifts in behavioral responses. Overall, changes in coping strategies in parents and children became more interconnected, indicating a change in stress response patterns for both.

In the pre-lockdown period, parental avoidance strategies were strongly negatively associated with a positive attitude, suggesting that parents who use avoidance coping were less likely to adopt a constructive, proactive view. However, we observed a change in this association in the post-lockdown period towards transcendence coping. This suggests that parents may have increasingly relied on spirituality or existential perspectives as a coping mechanism during the pandemic. This change in coping strategies could represent a psychological response to a situation of crisis, where individuals facing prolonged stress turn to transcendent beliefs for emotional regulation [25,26]. Moreover, we found a significant association between seeking social support and a problem-focused orientation among parents in the post-lockdown period of observation, suggesting that parents who actively sought external support were more likely to use constructive, solution-oriented coping strategies. This emphasizes the role of social networks in promoting adaptive coping responses, especially during crisis periods [27].

Children did not show a significant relationship among coping strategies before the lockdown but reported a stronger association between avoidance and aggressive behaviors in the post-lockdown period. Adoption of this strategy could develop through the psychological impact of social isolation, disrupted routines, and parental stress during lockdown, which could have contributed to maladaptive coping mechanisms in children [28,29].

The increased need for social support has become more associated in children during the post-lockdown, indicating evolving stress-related responses. Children’s avoidance behaviors showed stronger links with parental social support-seeking, while children’s coping strategies became more functional and were more associated with problem-focused and transcendent orientations. Moreover, we observed a decreased relationship between avoidance in girls and negative attitude in their parents. This shift suggests that as parents increasingly sought external support and problem orientation, their children may have adopted similar strategies, emphasizing the need for a supportive family environment during stressful periods. The decrease in maladaptive avoidance strategies post-lockdown aligns with previous research indicating that prolonged exposure to stress can lead to behavioral adjustments and adaptive coping mechanisms over time [27,30,31]. Besides, the observed increased association with transcendent orientation post-lockdown aligns with prior research emphasizing the role of spirituality in fostering optimism and reducing psychological distress in times of crisis, giving comfort [32,33].

Interestingly, geographical origin was found to be significantly related to the item “Transcendent orientation,” with parents of non-Italian origin scoring higher. Similarly, CCS questionnaire scores for girls showed a more functional coping attitude in facing early puberty both before and after lockdown, with a trend toward lower scores for “Avoidance” coping. This suggests a decreased use of this coping strategy in response to the emotional impact associated with the diagnosis of CPP after the lockdown. Moreover, additional analysis revealed that the “Avoidance” score appeared to be associated with both the COVID-19 pandemic and the geographical origin of participants, being higher in Italian girls compared to their non-Italian peers.

Previous studies have indicated an effect of geographical origin on spirituality in coping with the stress caused by disasters [34,35] and child illness [32]. People experiencing spiritual transcendence become more optimistic about life changes [33], and an increased transcendent orientation may support children with medical conditions by fostering a positive outlook, reducing psychological distress, and enhancing treatment adherence [32]. Also, origin differences could be observed when comparing child coping mechanisms. Children in the US preferred a more pragmatic, problem-solving-oriented attitude, whereas Nepalese children tended to resort more to coping strategies focused on the outbursts of emotions [36].

Overall, the use of adaptive coping strategies (problem-focused coping) versus maladaptive coping strategies (emotion-focused coping) has been found to be protective against the development of negative mental health outcomes (e.g., post-traumatic stress disorders, anxiety, depression, or substance abuse) for many children and adolescents who have experienced large-scale traumatic events [37,38] and adults [39]. Effective stress management by adults plays an important role in preventing children from using maladaptive coping strategies [40], which could explain changes during the post-lockdown period.

This study has several strengths, as it is the first study that compared coping strategies in girls diagnosed with CPP and their parents before and after a major psychological stressor. Only one previous study investigated overall coping in children with idiopathic central precocious puberty compared to premature thelarche [21] and found no significant differences in different strategy types in relation to these two diagnoses. Moreover, the use of validated psychometric tools enhances the reliability of the coping assessments, and the integration of clinical and psychological data provides a comprehensive analysis of the impact of the COVID-19 lockdown. 

However, there are some limitations. First, this is a retrospective analysis, in which subjects were asked to answer questions about their past, particularly the time of CPP diagnosis, which was a few years before. Second, potential factors, such as personality traits and mental well-being, that may be associated with coping styles were not specifically explored and should be considered in future research to better understand their role in shaping coping mechanisms. Next, the smaller sample size in the pre-lockdown group may have limited the statistical power to detect significant differences. Finally, the absence of long-term follow-up data post-lockdown limits any conclusions regarding the persistence of observed coping patterns over time.

## 5. Conclusions

The COVID-19 lockdown was associated with changes in functional stress-related coping mechanisms, which significantly evolved after the lockdown with increased reliance on social connections and problem-focused orientation, besides greater children’s emotional dysregulation. Avoidance strategies were initially prevalent in children with more negative support from parents pre-lockdown. However, there appeared to be a shift towards adaptive coping strategies such as problem-focused orientation and support-seeking coping mechanisms in children and their parents after the lockdown. Additionally, differences in coping strategies were observed in relation to cultural background, particularly with respect to transcendental orientation. These findings suggested that families may adjust their different behavioral patterns over time in response to prolonged stress. Therefore, parental strategies played an essential role in shaping children’s responses, highlighting the importance of family dynamics in managing stress associated with disease.

Based on our results, we emphasize the importance of addressing psychosocial factors in the management of CPP. Healthcare professionals should focus on developing problem-solving skills and promoting positive attitudes to counteract avoidance tendencies in children with precocious puberty and their parents, and support resilience in both children and their families.

Future research should explore the relationship between these coping strategies and biological markers of stress in children diagnosed with CPP.

## Figures and Tables

**Table 1:** Clinical features of girls diagnosed with CPP before and after the COVID-19 lockdown (M ± SD).

Variables	Pre-Lockdown *n* = 39	Post-Lockdown *n* = 135	Statistical Test	Effect Size	*p*-Value
Age, yr (at diagnosis) Weight, kg BMI SDS	8.04 ± 0.59	7.99 ± 0.88	U = 2535	rrb = 0.037	0.725
	33.31 ± 7.49	30.07 ± 7.27	U = 359	rrb = −0.248	0.119
	0.61 ± 1.08	0.03 ± 1.25	t = 1.930	Cohen’s d = 0.527	0.058
Height SDS Bone age, yr	0.83 ± 0.98	0.85 ± 0.97	U = 2473	rrb = 0.061	0.565
	9.25 ± 1.08	9.34 ± 1.28	U = 2213	rrb = 0.080	0.459
Delta CA—BA LH, IU/L LH peak, IU/L FSH, IU/L	1.31 ± 0.92	1.33 ± 0.72	U = 2237	rrb = 0.055	0.610
	1.08 ± 1.48	1.56 ± 1.88	U = 1878	rrb = 0.270	0.010 *
	15.47 ± 11.91	21.33 ± 14.19	U = 1802	rrb = 0.273	0.010 *
	3.34 ± 2.11	3.81 ± 2.24	U = 2218	rrb = 0.138	0.190
FSH peak, IU/L	12.49 ± 4.70	13.36 ± 4.57	U = 2132	rrb = 0.139	0.189
LH/FSH ratio	1.24 ± 0.72	1.88 ± 1.98	U = 1796	rrb = 0.275	0.010 *
Estradiol, pg/ml	44.18 ± 39.28	45.52 ± 43.57	U = 2027	rrb = 0.025	0.825
TSH, mU/L	2.23 ± 1.37	2.06 ± 1.16	U = 2129	rrb = −0.054	0.623
fT4, ng/dl	1.05 ± 0.16	1.27 ± 0.37	U = 1383	rrb = 0.138	0.249
Ovarian volume right, cm^3^	2.50 ± 0.82	2.67 ±1.24	U = 1888	rrb = 0.081	0.463
Ovarian volume left, cm^3^	2.33 ± 0.89	2.67 ± 1.37	U = 1678	rrb = 0.160	0.149
Uterine length, cm	36.16 ± 11.70	42.33 ± 8.16	U = 1135	rrb = 0.381	<0.001 *
Uterine body/cervix ratio	1.28 ± 0.45	1.70 ± 0.78	U = 181	rrb = 0.264	0.132

Note. BMI, Body Mass Index; CA, chronological age; BA, bone age; LH, luteinizing hormone; FSH, follicle-stimulating hormone; TSH, thyroid-stimulating hormone; fT4, free thyroxine; t, Student’s test; U, Mann–Whitney; rrb, rank biserial correlation. * *p* < 0.05.

**Table 2:** Coping strategies in children (CCS) and parents (COPE-NVI-25) before and after the COVID-19 lockdown (M ± SD).

Variables	Pre-Lockdown *n* = 39	Post-Lockdown *n* = 135	Statistical Test	Effect Size	*p*-Value
CSS					
CCS Avoidance	2.75 ± 0.43	2.64 ± 0.45	U = 3000	rrb = 0.140	0.180
CCS coping and support-seeking	3.01 ± 0.57	3.01 ± 0.44	U = 2732	rrb = 0.38	0.718
CCS aggressive behaviors	2.24 ± 0.70	2.28 ± 0.65	U = 2627	rrb = −0.002	0.985
COPE-NVI-25					
COPE positive attitude	4.31 ± 1.08	4.31 ± 1.13	U = 2610	rrb = −0.001	0.993
COPE seeking social support	4.68 ± 1.11	4.65 ± 0.97	U = 2824	rrb = 0.081	0.442
COPE problem-focused orientation	5.10 ± 0.99	5.07 ± 0.79	U = 2775	rrb = 0.062	0.555
COPE avoidance	1.53 ± 0.55	1.60 ± 0.60	U = 2406	rrb = −0.079	0.448
COPE transcendent orientation	2.71 ± 1.51	2.35 ± 1.26	U = 2896	rrb = 0.117	0.266

Note. CCS, Children’s Coping Strategies questionnaire; COPE-NVI-25, Coping Orientation to the Problems Experienced questionnaire filled out by the parents; U, Mann–Whitney; rrb, rank biserial correlation.

**Table 3:** Multiple linear regression analysis of predictors of CCS and COPE-NVI-25 in a subgroup of girls with central precocious puberty (*n* = 44).

Predictor	B	SE	95% CI	*p*-Value
CSS Avoidance				
Mother’s age at menarche	0.104	0.056	−0.011; 0.219	0.076
Non-Italian origin (vs Italian)	−1.099	0.244	−1.602; −0.596	0.002 *
Family history of CPP (yes vs. no)	−0.034	0.311	−0.675; 0.607	0.914
Adoption status (yes vs. no)	0.083	0.235	−0.401; 0.567	0.726
COPE transcendent orientation				
Mother’s age at menarche	0.113	0.193	−0.265; 0.491	0.564
Non-Italian origin (vs Italian)	1.829	0.845	0.858; 3.402	0.041 *
Family history of CPP (yes vs. no)	0.003	1.026	−2.008; 2.014	0.998
Adoption status (yes vs. no)	0.604	0.700	−0.768; 1.976	0.395

* *p* < 0.05.

## Data Availability

All data generated or analyzed during this study are included in the article. The data are fully available without restrictions, and inquiries can be directed to the corresponding author.
